# Protective Microbiota: From Localized to Long-Reaching Co-Immunity

**DOI:** 10.3389/fimmu.2017.01678

**Published:** 2017-12-07

**Authors:** Lynn Chiu, Thomas Bazin, Marie-Elise Truchetet, Thierry Schaeverbeke, Laurence Delhaes, Thomas Pradeu

**Affiliations:** ^1^University of Bordeaux, CNRS, ImmunoConcept, UMR 5164, Bordeaux, France; ^2^University of Bordeaux, INRA, Mycoplasmal and Chlamydial Infections in Humans, EA 3671, Bordeaux, France; ^3^Department of Hepato-Gastroenterology, Bordeaux Hospital University Center, Pessac, France; ^4^Department of Rheumatology, Bordeaux Hospital University Center, Bordeaux, France; ^5^Department of Parasitology and Mycology, Bordeaux Hospital University Center, Bordeaux, France; ^6^University of Bordeaux, INSERM, Cardio-Thoracic Research Centre of Bordeaux, U1045, Bordeaux, France

**Keywords:** host–microbiota symbiosis, colonization resistance, microbial ecology, disease tolerance, pathogens, containment, infectious diseases

## Abstract

Resident microbiota do not just shape host immunity, they can also contribute to host protection against pathogens and infectious diseases. Previous reviews of the protective roles of the microbiota have focused exclusively on colonization resistance localized within a microenvironment. This review shows that the protection against pathogens also involves the mitigation of pathogenic impact without eliminating the pathogens (i.e., “disease tolerance”) and the containment of microorganisms to prevent pathogenic spread. Protective microorganisms can have an impact beyond their niche, interfering with the entry, establishment, growth, and spread of pathogenic microorganisms. More fundamentally, we propose a series of conceptual clarifications in support of the idea of a “co-immunity,” where an organism is protected by both its own immune system and components of its microbiota.

## Introduction

The immune system is never at rest, nor is it ever isolated from its environment. It is in constant interactions with myriads of microbes, which can be pathogenic, commensal, or mutualistic (1–4). Recent work has shed light on the impact of commensal and mutualistic microbes on the development, induction, training, and functioning of the immune system [reviewed in Ref. (5–7)]. It is becoming increasingly clear that the microbiota, i.e., the microorganisms living persistently on and in a host, play a decisive role in shaping an effective host immune system and, *vice versa*.

Resident microorganisms do not just induce host immunity but can also directly inhibit pathogens (8–14). This phenomenon has been documented across many species—vertebrates, invertebrates, and plants (15, 16). The ability of commensals and mutualists to interfere with pathogen colonization and growth is known as “colonization resistance” (17–19). While the concept of colonization resistance is not new (Box 1), its underlying mechanisms are just being understood. Two main modes of protection have been proposed: resident microorganisms can protect the host either through *direct* microbe–microbe competition, which involves niche competition or direct antagonism, or by the *indirect* induction or priming of host metabolism or immunity, thanks to which the host is better protected against pathogen infections.

Box 1 **History of colonization resistance.**

Since the beginning of the antibiotics era in the 1950s, it became increasingly clear that indigenous microbial flora—generally assumed as the anaerobic autochthonous symbionts—can be a source of resistance against pathogens. Resistance conferred by the indigenous population explains the side effects of antibiotics (e.g., microbial overgrowth and superinfections) as well as the effectiveness of using antibiotics to prepare animal models for pathogen infection (20–22). The main mechanisms were thought to be competition between microbes, either over substrates or through bactericide molecule production (23, 24). When van der Waaij and colleagues (25) coined the term “colonization resistance,” they cited as possible mechanisms an indirect stimulation of gut movement in addition to direct antagonism between microbes, but rejected the involvement of the immune system. Yet, with the discovery of immunoglobulins (Ig) in the gut lumen and mucosa (26, 27), evidence for indirect, host-mediated mechanisms began to emerge: microbes were thought to assist the immune system by preventing Ig degradation or by significantly lowering pathogen load for Ig effectiveness (28, 29). In the meantime, proposals of direct mechanisms expanded (including competition over substrates and adhesion sites, release of antibacterial substances, prevention of translocation, and alteration of microenvironments to favor growth) (30–33). The 1980s and 1990s saw an explosion of research on colonization resistance and gut immune responses against pathogens, as well as the impact of bacterial flora on immune development (34). But only after the 2000s did breakthrough studies decisively demonstrate microbe-induced innate and adaptive immune responses, e.g., defensins (35), IgA specific to the mucosa (36), angiogenins (37), lectins (38), and modulated T-cell populations (39).

Colonization resistance constitutes an expanded sense of traditional protective immunity, as these mechanisms can in fact be considered part of the host’s defensive repertoire. Of course, the protective effects of microbiota components are context-dependent: some microbes can help protect the host in certain circumstances, but are detrimental in others. In light of recent studies, our main claim is that microorganisms, broadly construed, can exert a protective role, and that a key challenge is now to characterize the different ways this protection can occur.

There are two important gaps in the current literature on colonization resistance. First, current understanding of microbe-induced protection is generally limited to colonization resistance against pathogen establishment and growth. Yet the protection against pathogens also involves the mitigation of pathogenic impact without eliminating the pathogens, also called “disease tolerance” (40), and the containment of microorganisms to prevent pathogenic spread.

Second, the protective effects reviewed under colonization resistance are localized to the immediate vicinity of the protective microbes. Of date, little is known about the protective effects of microorganisms against pathogens in distal organs and tissue sites, even though it is established that microorganisms in one organ (mainly the gut) can influence immune responses in other organs (41). The relative lack of research on long-reaching microbiota-mediated protection in part arises from the assumption that microorganisms, once established within their niches, do not spread or move. Another reason could be the assumption that protective long-reaching effects are too weak to be clinically relevant, and thus, not significant enough for therapeutic purposes. Both assumptions, however, are questionable.

**Figure 1:**
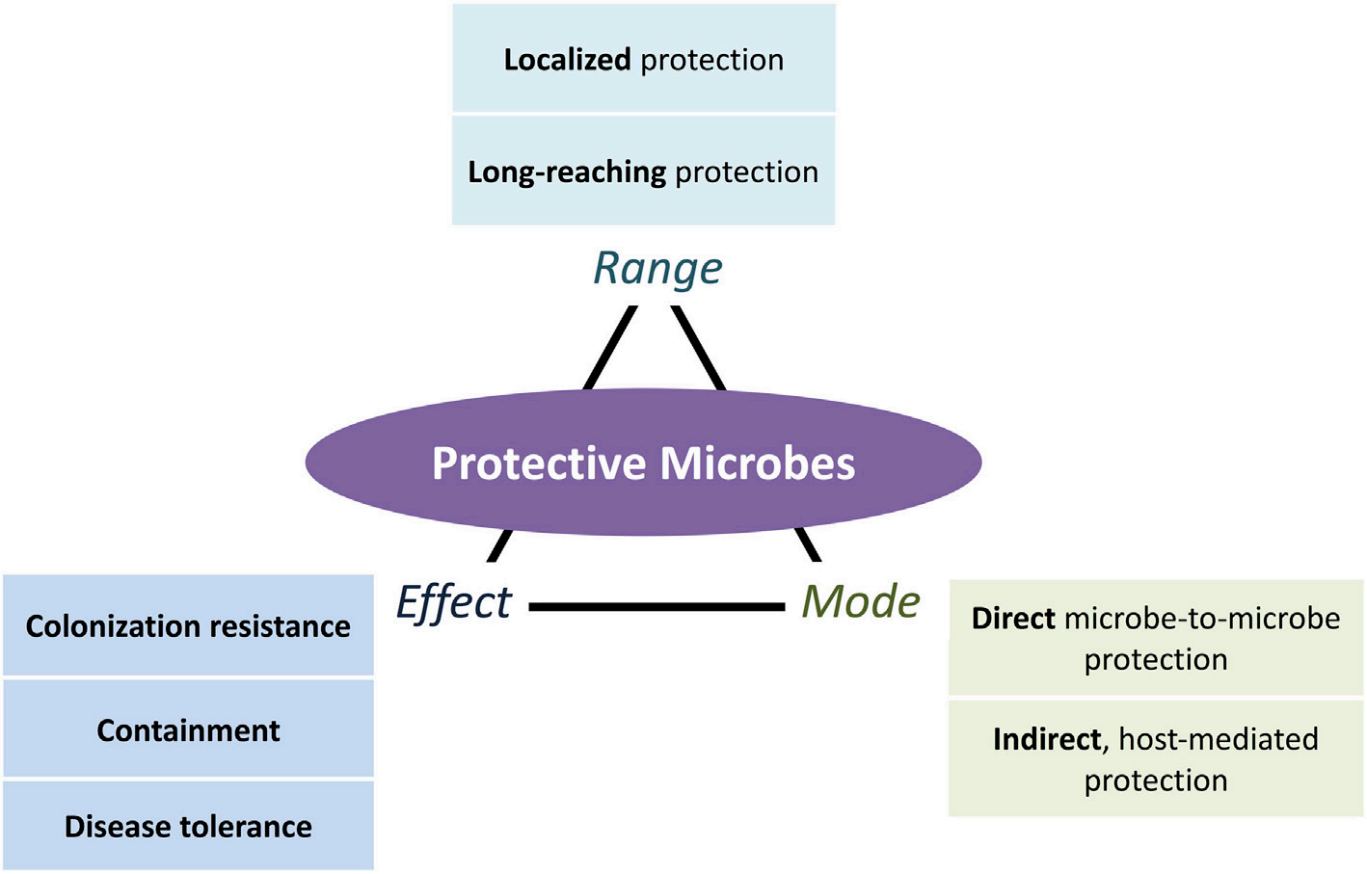
An expanded view of microbe-conferred protection. Microorganisms can protect the host in different ways, depending on the mode of protection (direct ecological and indirect host-mediated), the effects of protection (colonization resistance against pathogenic establishment and growth, containment of pathogens and their effects, and disease tolerance of pathogens while suppressing their negative effects), and the range of protection (localized or long-reaching, with the latter further divided into protection that is systemic or from one locale to another locale). All three aspects of protection can occur in combination. Long-reaching protection, for instance, involves both direct (ecological) and indirect (host-mediated) modes of protection that result in colonization resistance, containment, or disease tolerance effects.

In this review, we fill in the two gaps presented above with a systematic classification of protective mechanisms that include a broader range of defensive strategies differing in range (local, systemic, and long-reaching), mode (direct microbe-to-microbe and indirect host mediated), and effect (resistance, containment, and disease tolerance) (Figure 1). Addressing the first gap, we expand the effects of protection beyond colonization resistance to include containment of microorganisms and suppression of pathogenic impacts. We address the second gap by organizing existing evidence of the long-reaching of protective microbes.

Taking into account space constrains, our examples will focus only on vertebrates, with most of our examples coming from mammalian studies, especially humans and murine models [for example, in invertebrates, see Ref. (15)]. Because many of the terms used in this review have often been employed loosely in the literature and/or have been understood differently by different authors, here we explain in which sense we use them (Box 2). Finally, we offer important conceptual clarifications about the different ways components of the microbiota can exert protective effects on their host, and propose the concept of “*co-immunity*^1^” to describe the fact that host protection is in general the emergent and dynamic product of two influences, that of the host and that of some microorganisms (see Concluding Remarks and Perspectives).

Box 2 **Definitions.**

- *Immune protection against pathogens*: although immune responses can be very diverse, ranging from protection to development and repair (42), our focus in this article is on protection against pathogens. Consequently, our analysis excludes protection against immune diseases such as allergies, diabetes, and susceptibility to xenobiotics. We distinguish between direct, host-independent protection and indirect, host-mediated protection.
- *Microbiota*: when talking about the “microbiota,” we broadly consider all the resident microorganisms (microbes, but also “macrobes” such as helminthes) living in or on a host, regardless of the nature of their ecological interaction (parasitic, commensal, and mutualistic), size or taxonomy (parasites such as helminthes, fungi, bacteria, phages, or viruses). We also consider the synergistic and context-dependent effects of microorganisms.
- *Co-immunity*: a form of immune defense associating components of several organisms.
- *Colonization resistance*: host-dependent or independent resistance to pathogens that is induced by the microbiota. Current examples only concern the local inhibition of pathogen viability, establishment, or growth, but here we expand this concept to include long-reaching effects as well.
- *Containment*: controlled localization of microbes within a particular location inside the host body.
- *Disease tolerance*: limitation of host’s tissue damages induced by pathogens, without direct pathogen elimination.

## Localized Protection

In recent years, a general consensus on the mechanisms of colonization resistance has emerged (6, 9, 10, 18, 19, 43–46). Resident microorganisms can inhibit pathogenic viability and growth by antagonizing pathogens or “starving” them of limited resources, two well-recognized mechanisms of microbial ecological competition (47–49). In this section, we review expanded evidence that microorganisms confer local protection directly through microbe-to-microbe interactions (see Direct, Host-Independent Protection) and indirectly through the host immune system (see Indirect, Host-Mediated Protection) through colonization resistance, containment, and disease tolerance.

### Direct, Host-Independent Protection

#### Colonization Resistance: Beyond Niche Competition and Antagonism

Recent reviews have focused on a few aspects of colonization resistance. Protective microorganisms can antagonize pathogens through contact-dependent inhibition or the release of antimicrobial molecules. They can also outcompete pathogens for limited resources such as trace metals, nutrients, receptor donors, or adhesion sites, or construct environments hostile to pathogens, for instance, by lowering environmental pH (see summary of recent findings in Table 1). A recent finding, for instance, is that bacteria of the Clostridiales order outcompete *Listeria monocytogenes* for nutrition in the small intestine and likely antagonize the pathogen in other ways in the large intestine, providing the host a first line of defense from systemic infection (50).

**Table 1:** Types of colonization resistance.

Types	Species	Effects	Reference
Nutrition niche competition	*Bacteroides thetaiotaomicron* against *C. rodentium*	Competition for carbohydrates	(10)
	*Escherichia coli* Nissle 1917 against *Salmonella* Typhimurium	Competition for iron	(51)
	*Escherichia coli* HS and *E. coli* Nissle 1917 against EHEC O157:H7	Competition for carbohydrates	(52)

Antagonistic inhibition	*E. coli* Nissle 1917 against commensal and pathogenic *E. coli* and *S*. Typhimurium	Production of microcin	(53)
	*E. coli* against EHEC O157:H7	Production of colicin	(54)
	Nasal *Staphylococcus lugdunensis* against *Staphylococcus aureus*	Production of lugdunin	(55)
	*Enterococcus faecalis* strain against *Enterococcus*	Production of bacteriocin (pPD1)	(56)
	*B. thuringiensis* against *Clostridia* species	Production of bacteriocin (thuricin CD)	(57, 58)
	*Bacillus amyloliquefaciens* against vaginosis-associated human pathogen *Gardnerella vaginalis*	Production of bacteriocin (subtilosin)	(59)
	*Staphylococcus epidermidis* peptides selectively reduce survival of *Streptococcus pyogenes* and *S. aureus*	Production of phenol-soluble modulins (PSM-γ and PSM-δ)	(60, 61)
	Four bacterial consortium (*R. gnavus* E1, *B. thetaiotaomicron* LEMF4, *Clostridium hathewayi* LEMC7, and *Clostridium orbiscindens* LEMH9) against *Clostridium perfringens*	Collective production of consortium-dependent antibacterial substance	(62)
	Against EHEC O157:H7	Production of short chain fatty acids (acetic, propionic, and butyric acids)	(63)
	Against *Clostridium difficile*	Production of secondary bile acids	(44, 64–66)
	*Bacteroides fragilis* against *Bacteroidales* strains in gut	Type IV system delivered toxins	(67, 68)
	Lactic acid bacteria against a range of pathogens	Lactic and acetic acid, metabolites (hydrogen peroxide and carbon dioxide), diacetyl, and bacteriocins	(69, 70)

Niche construction of disadvantageous environments	Lactic acid bacteria in vagina against bacteria and viruses	Lower environmental pH with lactic acid	(71)
	*Propionibacterium acnes* suppresses the growth of *S. aureus*	Lower environmental pH with fermentative products	(72)
	Anaerobic commensals against *Enterobacteriaceae*	Low oxidation–reduction potential	(73)

The concept of the ecological niche is central to colonization resistance. Protective microorganisms are thought to “defend” their nutritional niche by killing incoming pathogens or outcompeting them (74). Pathogens, on the other hand, are thought to overcome colonization resistance by creating or exploiting new spatial-temporal niches (19). However, a niche is not just a nutritional environment, but any environmental feature relevant for colony survival and growth. Protective microbes can also inhibit pathogenic establishment and growth by disrupting pathogenic biofilms, bacterial collectives that undergo regular developmental “life cycles” and protected by synthesized extracellular polysaccharides matrices. Proteases released by *Staphylococcus epidermidis* can degrade the matrices of pathogenic *Staphylococcus aureus* biofilms (75, 76). Lactic acid bacteria can disrupt matrix synthesis by interfering with pathogen virulence genes, for instance, by decreasing the expression of *Streptococcus mutans* genes involved in matrix glucan production (77) or modulating *S. aureus* gene expressions related to the production of intercellular adhesion polysaccharides (78).

Protective microorganisms can also influence quorum sensing, the cell-to-cell communication system that allows bacteria to perceive information about bacterial population density and to regulate collectively virulence factor production and biofilm development (79, 80). Soluble molecules released by probiotics can interfere with the *S. aureus* accessory gene regulator (*agr*) quorum sensing system, which regulates the switch between biofilm and free-floating lifestyles (81). Subtilosin, a protein secreted by *Bacillus subtilis*, also interferes with the quorum sensing of *Gardnerella vaginalis*, preventing biofilm formation (82). Biosurfactants are well-known anti-adhesion, anti-biofilm agents that can also disrupt cell-to-cell signaling (83, 84). Cell-bound biosurfactants of lactic acid bacteria disrupt the biofilms of multi-drug-resistant *Acinetobacter baumannii, Escherichia coli*, and *S. aureus* (85) as well as *Serratia marcescens* strains (86).

#### Beyond Colonization Resistance: Containment and Disease Tolerance

Colonization resistance is not the only way microorganisms can protect the host from pathogens, as not all types of protection act by inhibiting pathogenic colonies establishment and growth. The role of resident microorganisms in barring entry and dispersal of alien populations is oftentimes ignored (87). Furthermore, inhibition of quorum sensing can affect pathogenicity, the effects of pathogens, without inhibiting bacterial viability, which, as a therapeutic option can avoid the selection of drug-resistant bacteria (88). We thus propose looking beyond colonization resistance to adopt a community ecology point of view that better understands the multiple ways resident communities can prevent or disrupt pathogen invasion.

In invasion ecology, residents can disrupt alien invasion by intervening with alien entry and diversification, by inhibiting its establishment or growth, by mitigating its negative impacts on local communities, or by preventing alien dispersal into other vulnerable environments (89–91) (see summary of stages of invasion in Table 2). A more comprehensive picture of resistance against pathogens must take into consideration the multiple stages of invasion and go beyond competition as the only way residents can fight against invaders.

**Table 2:** Stages of ecological invasion.

Four stages of ecological invasion	Entry		Establishment and growth			Negative effects	Spread
Seven sub-stages	1. Transportation: dispersal of microorganisms to a new sit	2. Introduction: entry into a new site	3. Establishment: survival and self-sustaining population	4. Growth: expansion	5. Diversification: mutation, lateral gene transfer, or adaptive evolution	6. Impact: negative effects on local communities	7. Spread: dispersal beyond the site of introduction
Macroecology, synthesis of plant, and animal ecology (92)	Transportation	Introduction	Colonization and naturalization			Impact	Spread

Macroecology, synthesis of plant, and animal ecology (93)	Transportation	Introduction	Establishment				Spread

Microbial ecology, based on plant ecology (90)	Introduction (passive or active dispersal beyond abiotic barriers)	Establishment (resource competition against biotic barriers)	Growth and spread (access to local resources and niche construction, adaptive evolution, and horizontal gene transfer)			Impact (following displacement or alteration of community function)	

Microbial ecology, based on community ecology principles (87)		Entry	Establishment		Diversification		

Microbiome ecology, based on plant ecology (89)	Dispersal	Colonization	Establishment	Spread			Spread

**Figure 2:**
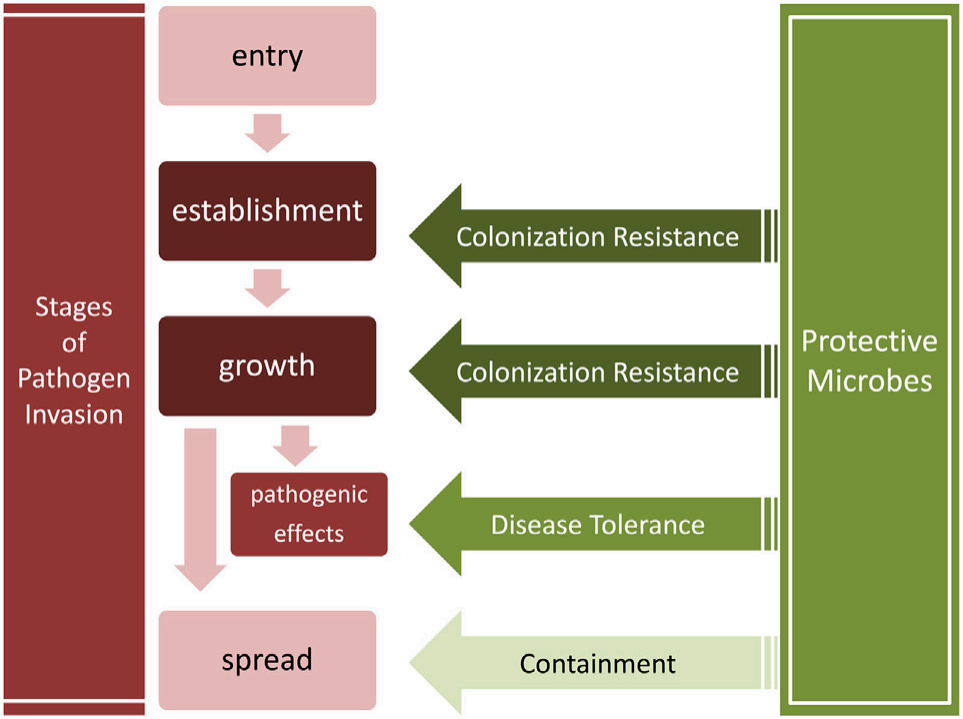
Stages of pathogen invasion and the obstacles presented by resident communities. Colonization resistance, containment, and disease tolerance present obstacles to different stages of the invasion process. Colonization resistance disrupts the establishment and growth of pathogens, the containment of pathogens prevents their spread into other tissues, and disease tolerance suppresses the negative effects of pathogens without decreasing their load.

We thus distinguish three major microbial obstacles against invading pathogens (Table 2; Figure 2): *colonization resistance* exclusively refers to the prevention of pathogen establishment of a persistent colony and population growth, *containment* is the prevention of pathogen spread into another body site, sometimes leading to systemic infection, and finally, *disease tolerance* is the limitation of pathogenic impact on host tissues without killing the pathogens.

*Containment* is the controlled localization of microbes and their effects within a particular location inside the host body. One example is the intestinal lumen. The epithelium barrier and gut mucosal immune system prevents the translocation of microorganisms from the lumen into the host. Mucosal-associated microorganisms have long been postulated as reinforcers of gut barriers, both directly and indirectly through the host (94–97). For instance, in various organisms, bacteriophages that adhere to mucus glycoproteins can prevent translocation of bacteria, thus providing a host-independent protection of mucosal surfaces against bacterial infections (98). *Saccharomyces boulardii* also prevents *Salmonella* liver translocation by directly binding to the pathogens (99).

Secreted factors can interfere with the translocation of gut pathogens into and beyond the epithelia. Supernatants of *Enterococcus mundtii* and *Lactobacillus plantarum* inhibit the invasion of *L. monocytogenes* into epithelial cells (100). A secreted, non-bacteriocin component from *Escherichia coli* Nissle 1917 also reduces the efficiency of *Salmonella enterica* serovar Typhimurium epithelial invasion and blocks invasion by many pathogens without eliminating them (101). Pathogen translocation can also be prevented by interference with pathogen adhesion to the epithelium (102, 103). It is well known that resident commensals, especially lactic acid bacteria, compete with pathogens for adhesion sites, for instance, the competition between *Lactobacillus reuteri* and enteropathogenic *E. coli* (104). Short chain fatty acids are products of anaerobic intestinal microbiota fermentation of dietary fibers. Butyrate inhibits the virulence genes that enable intestinal *S. enterica* subspecies to enter the epithelium, specifically pathogenicity island 1 (105). Acetate from *Bifidobacteria* can enhance epithelial cellular defense functions, inhibiting the translocation of Enterohemorrhagic *E. coli* (EHEC) shiga toxin into the bloodstream (106).

*Disease tolerance*, on the other hand, is the limitation of pathogen-induced damage of host tissues without elimination of the pathogen. Various types of processes are associated with disease tolerance: the neutralization or inhibition of pathogenic toxins, the healing process of the host, and the regulation of destructive inflammation and maintenance of metabolic homeostasis (see Box 3). While the latter two are mediated through the host, the first can be host-independent.

Box 3 **The concept of disease tolerance.**

Disease tolerance should be distinguished from “tolerance” or “immune tolerance,” a low or regulated immune reaction toward an antigen (40). It is instead the limitation of pathogenic damage to host tissues. The concept of disease tolerance has long been recognized by plant ecologists as an alternative mode of protection from pathogens or pests besides resistance mechanisms, yet only relatively recently applied to animals (107–110). Even though disease tolerance is currently seen as an indirect process mediated by the immune system to control tissue damage (40), it could be directly regulated by microbes as well. The toleration of pathogens could be a cost-effective alternative when preservation of pathogen fitness does not strongly decrease host fitness (e.g., when pathogen virulence is low), especially since the side effects of pro-inflammatory immune responses are sometimes more damaging to the host than the infections themselves.

An example of host-independent disease tolerance is the ability of resident microbiota to inhibit gene expressions of toxins. Soluble molecules from *Bacteroides thetaiotaomicron* and the bacteria-produced short chain fatty acid butyrate, for instance, can inhibit EHEC shiga toxin gene expressions and pathways (111, 112). The quorum sensing molecule AI-2 of *Ruminococcus obeum* also downregulates the *Vibrio cholera* toxin operon (113). Secreted organic acids can mitigate pathogen toxicity in various ways: lactic acid from *Streptococcus thermophilus* inhibits *Clostridium difficile* toxin A gene expression (114) while acetic acid secreted by a Bifidobacteria strain inhibits EHEC shiga toxin production in part by lowering environmental pH (115). Remarkably, the growth of shiga-producing *E. coli* was not inhibited by the *B. breve* strain even though toxin production was halted.

The damaging effects of pathogens can also be diminished by the degradation of released toxins. Proteases secreted by a probiotic *Bacillus clausii* strain acts against toxins from *C. difficile* and *Bacillus cereus* (116). Another example is a *Saccharomyces boulardii* protease that inhibits *C. difficile* toxin A and B (117). Bifidobacteria and Lactobacilli strains mitigate *C. difficile* cytotoxic effects by inactivating their secreted toxins (118, 119).

**Figure 3:**
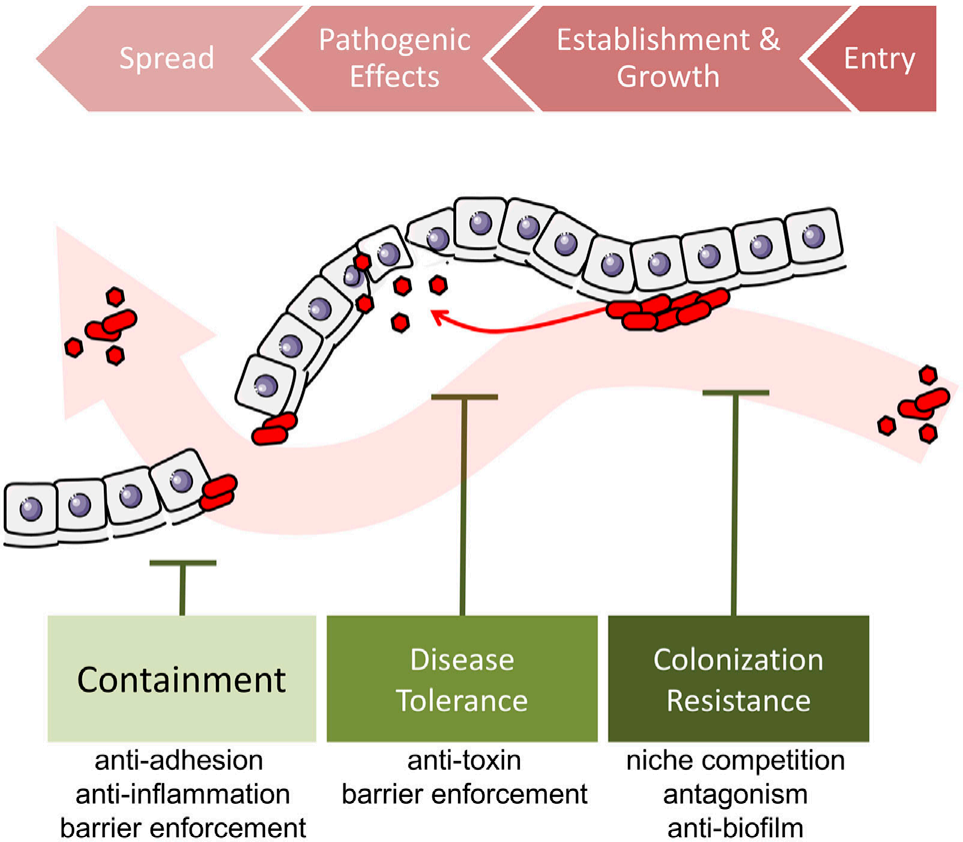
Localized microbe-conferred protection against pathogen invasion. Alien pathogens invade the host by entering a host site, establishing a growing colony, exerting negative effects on the host. When conditions are ripe, they can spread to a different host site, in this case, through the epithelium or endothelium barrier. Protective microorganisms can challenge pathogen invasion at any of the four stages, by disrupting entry, preventing or destroying colony establishment and growth, by suppressing pathogenic effects, or by preventing spread into other tissues.

**Figure 4:**
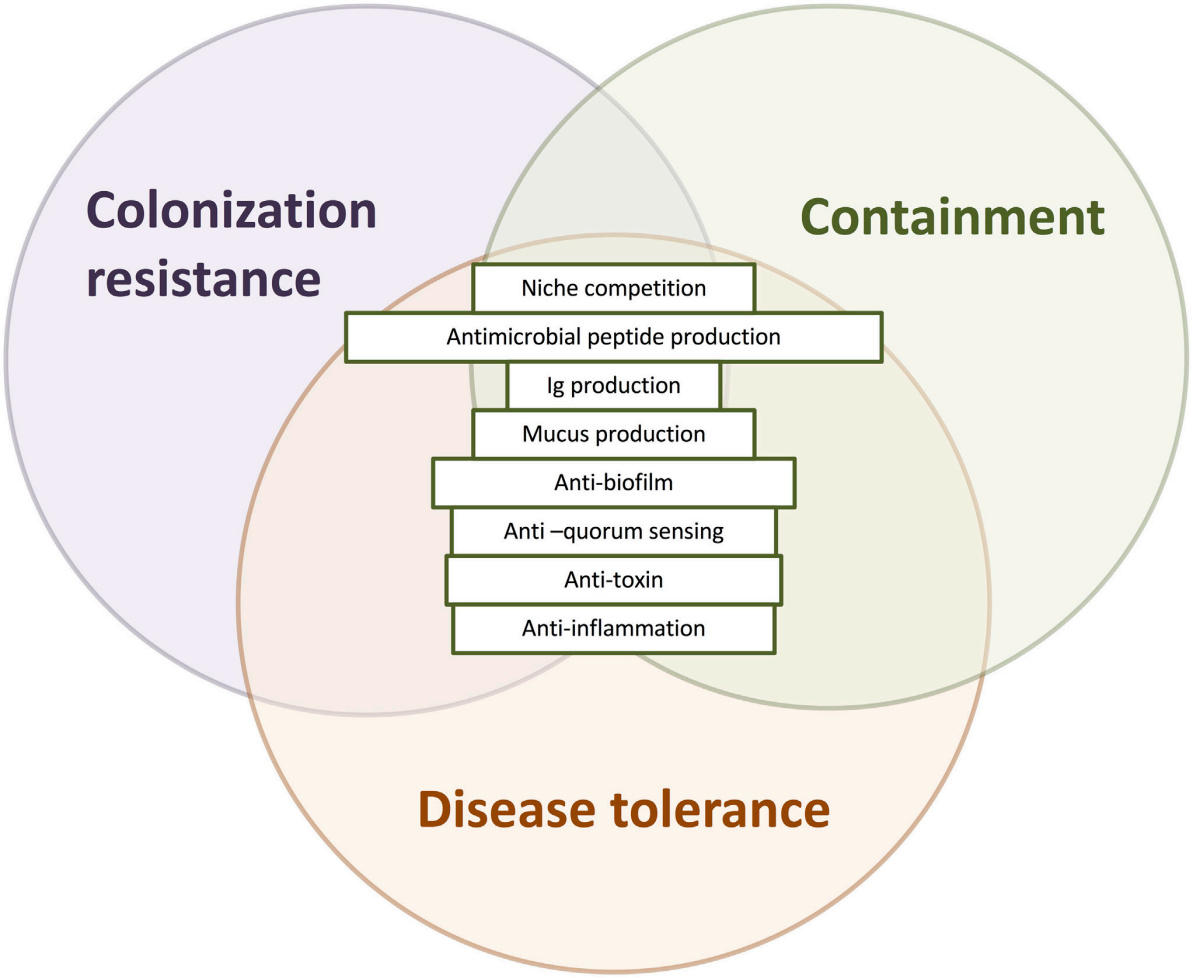
The overlapping mechanisms of colonization resistance, containment, and disease tolerance. Colonization resistance, containment, and disease tolerance are distinct effects on invading pathogens, disrupting their growth and establishment, their spread, and their negative effects, respectively. Nevertheless, the mechanisms underlying these effects can overlap, deployed at different times and stages with different outcomes. Antimicrobial peptides, for instance, prevent the translocation of microorganisms as part of the mucus barrier and disrupt the establishment and growth of pathogens. Another example is the disruption of a pathogenic biofilm, which may destroy the establishment of the colony as well as prevent it from adhering and translocating gut lumen.

In sum, colonization resistance, containment, and disease tolerance are diverse effects of protective microbes against the spread, establishment and growth, and impact of pathogen invasion. Broadening the concept of protection to include the effects of disease tolerance and containment allows us to consider pathogenicity in a wider context, taking into account the overall ecological process of pathogen invasion (Figure 3). The detailed mechanisms underlying these three types of effects—colonization resistance, containment, and disease tolerance—may overlap. For instance, competition over adherence sites prevents the growth of biofilms that enable the establishment, growth, and spread of many pathogens (Figure 4).

The examples reviewed so far are direct microbe-to-microbe effects. We now turn to indirect forms of protection that involve host systems.

### Indirect, Host-Mediated Protection

Local microbiota-induced protection of the host can also be indirect, that is, mediated by host immunity and metabolism. We will focus on protection that involves the host immune system. Microorganisms induce proper development of crucial components of the host immune system, such as mucosa-associated lymphoid tissues (120–122). Here, we will more precisely focus on microbe-induced host immunity that specifically acts against pathogens (4, 123), guarding against both harmful (pathogens) and potentially harmful (“pathobionts”) microorganisms.

#### Colonization Resistance

Examples of host-mediated protection, especially colonization resistance, by gut microbiota have already been extensively reviewed (see Box 1). Indirect colonization resistance occurs when resident microorganisms induce host reactions that act against pathogens.

Resident microbiota sampled by pattern recognition receptors can activate downstream secretion of antimicrobial peptides that protect the host by killing or inactivating bacteria, fungi, and viruses, primarily by destroying cellular membranes (124, 125). These include C-type lectin Reg3-γ (38), α and β defensins (35, 126), and the ribonuclease angiogenin (127). The effects are often pathogen- and host-specific (128–130).

Gut microbes induce the development of lymphoid tissues that sample and secrete Ig A into the gut lumen as well as regulate secretory IgA (SIgA) secretion levels (131). SIgA can help trap and exclude pathogens (“immune exclusion”), but when bound to certain microorganisms, can also selectively promote commensal biofilms that confer colonization resistance against pathogens (“immune inclusion”) (132–136). Specificity to microorganisms could be enhanced through a positive feedback loop mediated by SIgA retrotransportation (137–139).

Microbes can also induce protective pro- or anti-inflammatory immune activities by altering the balance between host T-cell subsets (2, 140–142). By tilting the balance toward pro-inflammatory pathways, commensals help generate host-mediated attacks against pathogens. Segmented filamentous bacteria, for example, induce inflammatory Th17 immunity (143), leading to a generally protective, although context-dependent immune state (144). Protozoan *Tritrichomonas musculis* protects from gut *S*. Typhimurium infection through promotion of inflammatory Th1 and Th17 type immunity (145).

In the skin, microbiota could be considered an “endogenous adjuvant” of the skin immune system, exerting its influence *via* the release of products (such as antimicrobial peptides or metabolites) and/or *via* the modulation of innate and adaptive immunity without invoking inflammatory responses (146–148).

In the respiratory tract, local manipulation of the microorganism composition may have profound consequences on the capacity of the host to mount protective responses (149). For instance, intranasal inoculation of mice with live or heat-inactivated *Lactobacillus* spp. and non-pathogenic *Listeria* spp. protects against secondary lethal infection with the virulent pneumonia virus by suppressing virus-induced pro-inflammatory cytokines and viral load (150).

#### Beyond Colonization Resistance: Containment and Disease Tolerance

Microbes can also indirectly protect the host by containing pathogens within the gut and lung lumen, as breaching of the epithelial layer triggers systemic inflammation. New developments shed light on the intimate relation between microorganisms and barrier functions. The gut epithelium barrier is fortified by maternal microorganisms before birth (151). After exposure to environmental microbes, microorganisms strengthen and protect the barrier (152). *In vitro* treatment of *Saccharomyces boulardii*, for instance, prevents *Bacillus anthracis* toxins from destroying the integrity of epithelium cells and the tight junctions between them (153). In antibiotics-treated mice, translocation of bacteria and an increase in inflammatory responses can be observed (154, 155).

The separation of host and microorganism with a mucus layer is in part regulated by resident microorganisms. The mucus layer effectively separates luminal components, especially microorganisms, from the epithelium. The constant outgrowth of mucus pushes microorganisms out and provides a medium that concentrates antimicrobial elements such as antimicrobial peptides and SIgA. In the small intestine, Muc2 mucin, the backbone of mucus in mice, requires bacterial cleavage of a proteolytic enzyme to detach mucin from secreting goblet cells; in the colon, “sentinel” goblet cells activated by bacterial components quickly respond by extruding and releasing an explosion of Muc2 (156, 157). Helminthes such as *Trichuris muris* can restore the Nod2-deficient abnormal goblet cells of mice through T_H_2-mediated immunity, thereby inhibiting the colonization of pro-inflammatory *Bacteroides* species (158).

Localized protection is also achieved by disease tolerance. In the gut, *Bacteroides fragilis* affects systemic T-cell responses through the action of the bacterium-derived polysaccharide A, which protects against pathobiont *H. hepaticus* colitis *via* the production of anti-inflammatory IL-10 by CD4+ T cells and the promotion of regulatory T cells (159). Moreover, this protection has no effect on pathogen fitness or on population load in the intestine. SIgA can neutralize intracellular toxins and viruses, as well as directly suppress bacterial virulence mechanisms (133, 135).

In sum, resident microbiota can protect the host by locally inhibiting the establishment and growth of pathogens, mitigating pathogen-induced damage to the host, and preventing the spread of microbes to other sites. Local protection is ensured by mechanisms that are directly targeted against microorganisms or indirectly through the host immune system.

## Long-Reaching Protection

We now turn to protective microbes that help the host from a distance, by either inducing systemic protection (that is, a protection realized in the entire organism) or protection at a particular remote site. In host-microbiota symbiosis, the habitat of the microbial communities is a changing and responsive living being. Since host microbial communities are connected by metacommunity dispersal and through the internal sensors and networks of the host, protective microbes can have potentially far-reaching and integrated consequences. A more comprehensive picture of protective microbes requires a global understanding of the ecological and physiological—e.g., metabolic, immune, and neuroendocrine—interactions.

**Figure 5:**
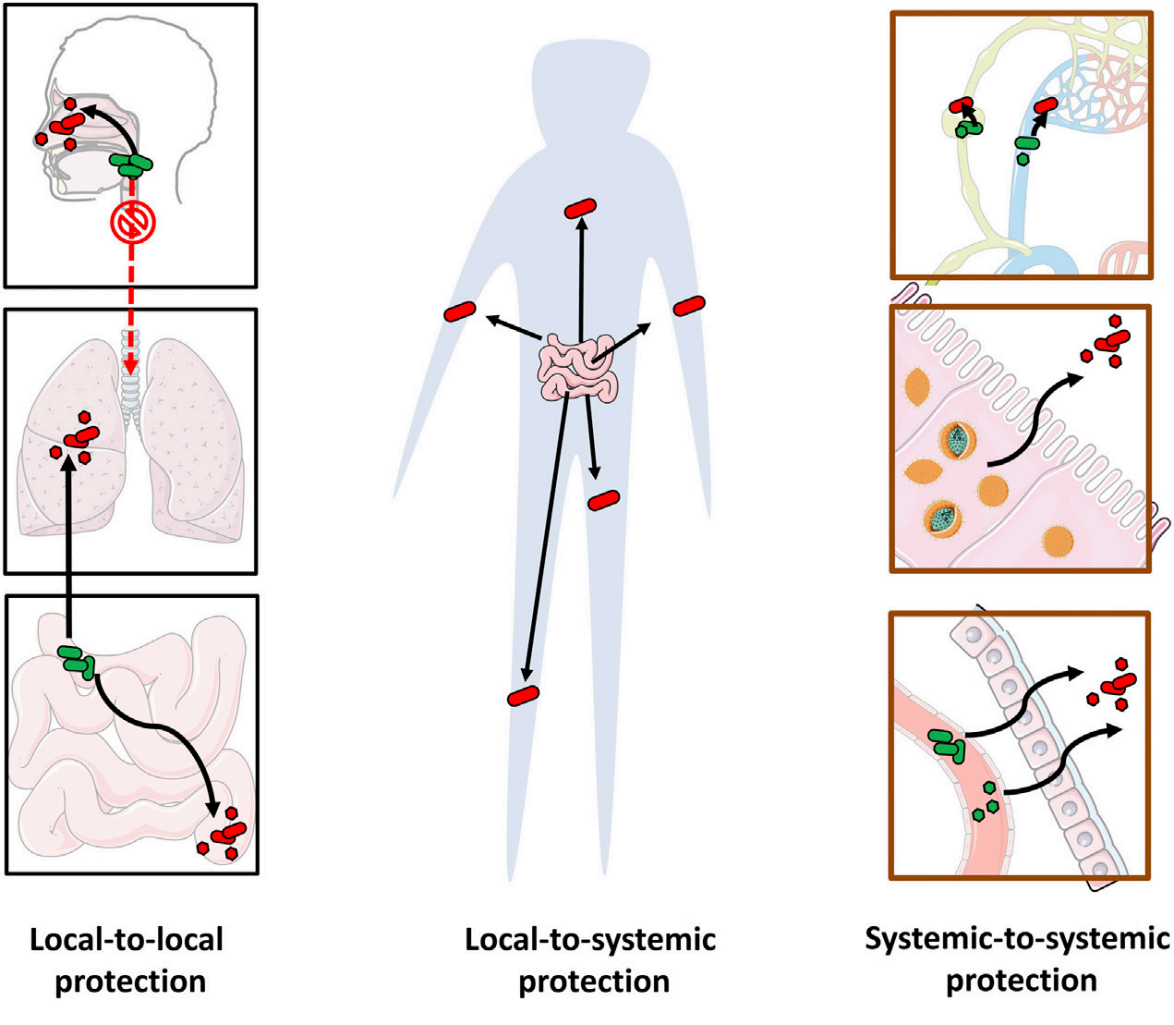
Long-reaching microbe-conferred protection against pathogens. The multiple pathways of long-reaching protection include: local-to-local protection against pathogens at a distal site by protective microorganisms at another site (e.g., gut to lung, upper respiratory tract to lower respiratory tract, and small intestine to large intestine) (left), local-to-systemic protection from one site to pathogens across the body (e.g., systemic protection from gut microbiota) (center) and systemic-to-systemic protection by microorganisms that are distributed systemically (e.g., protective viruses or bacteria that circulate through the blood stream or the lymphatic system, or reside in multiple locations) (right).

However, our understanding of long-reaching protection is at its infancy. Whereas pathogens can migrate through the blood from one organ to another or travel across microenvironments within an organ system (160–162), there is little evidence that commensals can do the same. Here we review examples of long-reaching protection (see Figure 5).

### Systemic Protection

The direct, host-independent systemic effects of microorganisms are poorly illustrated in the literature, and as far as we know, there are no mammalian examples where microorganisms can confer direct systemic protection across microhabitats. Nevertheless, the killing capacities of some microorganisms present promising therapeutic options as “live antibiotics.” For example, the predatory proteobacteria *Bdellovibrio bacteriovorus*, when injected in a zebrafish model infected with *S. flexneri*, preys and eliminates the human pathogen in the whole animal (163).

Indirectly, however, the evidence is much more promising as microorganisms have far-reaching immunomodulatory effects on mucosal and systemic immunity (164, 165). Microbiota modulation of myeloid cells preconditions neutrophils induces macrophage killing capacities and calibrates macrophages and dendritic cell responsiveness to infections (166). Microbial-regulated hematopoiesis can fight against systemic infections such as *L. monocytogenes* (167). In a mice model, injection of chitin from the fungal commensal *S. cerevisiae* increases resistance to systemic infection with *C. albicans* by inducing monocyte activity *via* a fine modulation of TNF-α and IL-6 (168). Without the microbiota, mononuclear phagocytes fail to prime NK cells that normally trigger systemic antiviral immune responses in non-mucosal lymphoid organs (169). Recent studies show that maternal microbes shape offspring immunity before birth. Bacterial products in mothers induce genetic expression of antimicrobial peptides in offspring epithelium and shape the components of their innate immune systems (170) while dampening their adaptive immune responses (171). In fact, the success of vaccination (the injection of microbe-derived products in one site that protect against infections in another site) shows that long-distance immune protection is possible. For instance, the intranasal administration of a vaccine against HSV-1 induces production of specific antibodies in the genital tract (172).

While current evidence is still not clear-cut, it is nevertheless important to conceptually distinguish between various *origins* of systemic protection. In local-to-systemic protection, microorganisms at a single location, such as the gut microbiota, can confer protection through systemic immune responses or leave the local site and disseminate to other regions. In systemic-to-systemic protection, the protective microorganisms are already distributed systemically, exerting system-wide localized effects. In particular, we discuss how microbiota, their fragments, or by-products can reach distal sites through the host circulatory systems.

#### Local to Systemic Protection

Microbiota at one location can induce systemic colonization resistance in the host. Composition of the gut microbiota modulates the severity of malaria, where a fecal transplant from resistant mice transfers the resistance to germ-free mice by elevating humoral immune responses. The resistant gut microbiota are characterized by an increased abundance of *Lactobacillus* and *Bifidobacterium* (173). In humans, gut microbe *E. coli* O86:B7 induces antibodies that target plasmodium sporozoites, conferring a protective cross-response against malaria transmission (174).

Systemic innate and adaptive immune responses to *Toxoplasma gondii* infection in humans rely on the indirect stimulation of dendritic cells by normal gut microflora (175). Several studies have shown that gut microbiota also have the potential to reduce systemic viral infection and disease (176). A clinical trial observed a positive effect of a *Lactobacillus* strain as an immune adjuvant for live-attenuated H3N2 influenza vaccine in healthy adults, with higher seroconversion rates in patients treated by probiotics (177), suggesting a non-specific immune response activation. In HIV infection, higher abundance of the gut Lactobacillales order in patients is positively associated with CD4+ T cell count and negatively associated with viral load, indicating that bacteria from Lactobacillales could in some direct or indirect way modulate the infectivity or pathology of HIV infection (178).

Finally, as a basal stimulant, bacteria can prime activation threshold of antiviral innate immunity against systemic viruses (179). Antibiotic-treated mice before lymphocytic choriomeningitis (LCMV) infection elicited an impaired innate and adaptive immune response to LCMV infection, and an increased mortality after influenza infection (179).

#### Systemic to Systemic Protection

Microbiota or its components that are normally distributed across the host system can also induce system-wide colonization resistance in the host. Recently, the idea that viruses can be mutualistic, and in particular can offer protection against pathogens, has gained popularity (180–182). A healthy human is infected by more than 10 permanent chronic systemic viruses, and this number may in fact be far higher (182). Two cases show that viruses induce in their hosts a higher basal immunity that explains normal immunity as well as responses to novel microbes. First, baseline activities can be upregulated and maintained by viruses in latent states, conferring protection against subsequent infections (176, 183). In mice, gammaherpesvirus 68 or murine cytomegalovirus (CMV) (which are models for the human pathogens Epstein–Barr virus and CMV, respectively) in dormancy confers symbiotic protection against bacterial infection to *L. monocytogenes* and *Y. pestis* in an antigen-independent way, involving interferon production and macrophages systemic activation (184, 185).

In a clinical study, HIV patients co-infected with non-pathogen GB flavivirus showed reduced mortality rate, suggesting the possibility of an inhibition of HIV replication due to this flavivirus (186). HIV replication was reproducibly inhibited in cultures of peripheral-blood mononuclear cells by GBV-C coinfection (187). Patients with GBV-C infection present an increased proportion of naive T cells and a reduction in T-cell activation and proliferation that could increase disease tolerance and finally the survival among HIV infected individuals co-infected with GBV-C (188).

#### Circulations and Protective Effects

It is generally assumed that microorganisms are pathogenic when spread throughout the system while non-pathogens are contained within the gut and lung lumens and outside of skin surfaces. Indeed, bacteremia and endotoxemia, i.e., the spreading of bacteria or bacterial structural components through blood circulation, respectively, are usually pathological. For instance, intra-uterine infection during pregnancy in mice can be caused by pathogens that do not belong to vaginal microflora, but to oral microorganisms *via* hematogenous transmission (189).

However, non-pathological, physiological blood circulation of microorganisms or microbial fragments occurs in healthy subjects (190–192). Substantial numbers of live gut species have also recently been found living in the mesenteric lymph nodes and systemic lymphoid organs under normal situations (164). In this section, we thus consider the possibility that circulation of bacteria or their metabolic compounds can constitute a long-reaching route of protection. Even though a long-reaching seeding through the bloodstream and lymphatic systems has never been demonstrated, it is a possible route protective microbes could take.

The involvement of circulating long-reaching mechanisms in host protection is only a hypothesis at this stage, but investigating this potential long-reaching influence undoubtedly constitutes a very promising avenue for future research, paving the way to targeted experiments.

##### Bacteremia and Endotoxemia

Bacteremia is the dissemination of bacteria into the circulatory system. Under normal conditions, Gram-negative bacteria in the gut can disseminate systemically, inducing system-wide production of IgG that provide cross-protection against Gram-negative bacteria infections such as *E. coli* and *Salmonella* (193).

Recent data suggest a long-distance disease tolerance by challenge-induced translocation of gut microbiota to multiple distal sites. In mice, infection with the respiratory pathogen *B. thailandensis* (intranasally) or with the pathogen *S*. Typhimurium (orally) leads to skeletal muscle wasting. Gut commensal *E. coli* O21:H+ antagonizes muscle wasting during these infections, with no changes in inflammation profile but sustained insulin-like growth factor 1 signaling in skeletal muscle. This protection was associated with the translocation of *E. coli* O21:H+ from the intestine to white adipose tissue and occurred without a negative impact on *B. thailandensis* or *S*. Typhimurium fitness. This beneficial effect promoted both the health of the host and fitness of *E. coli* O21:H+ (194).

Endotoxemia occurs when bacterial by-products circulate into the bloodstream. It has long been known that circulating lipopolysaccharides, a membrane component of Gram-negative bacteria, has potentially beneficial immunomodulatory effects (195). Lipopolysaccharides endotoxemia is increased by nutritional factors (196) and modulated by changes in gut microbiota (197). The mechanisms of host-mediated modulation of bacteremia and endotoxemia (primarily of lipopolysaccharides) are currently being explored under the context of high-fat intake or obesity, which weaken gut integrity leading to increased penetration of gut microbes or their products into the circulation. The uptake and transportation of lipopolysaccharides is active, reaching from the gut to distant tissues like adipose tissue. After lipoprotein binding and transportation to the lymph and across endothelial barriers, they interact with macrophages and trigger the secretion of pro-inflammatory cytokines (198).

Microbiota-derived peptidoglycan translocated from the gut to neutrophils in the bone marrow systemically primes the innate immune system, enhancing the elimination of two major pathogens, *S. pneumoniae* and *S. aureus via* the pattern recognition receptor nucleotide-binding, oligomerization domain-containing protein-1 (199).

Finally, another active long-reaching route is the gut–liver axis, where influx of microbial molecules derived from genetic inflammasome deficiency-associated dysbiosis, passing in portal circulation, can trigger liver inflammation through TLR4 and TLR9 activation. In the case of preexisting lipid accumulation in hepatocytes, this mechanism could lead to non-alcoholic steatohepatitis (200).

##### Metabolic Compounds

Naïve mass spectrometry-based metabolomics studies comparing blood metabolic profiles between germ-free mice and conventional animals show a drastically different blood metabolic profile, with a drug-like metabolic response of the host to metabolites (i.e., degradation of xenobiotic molecules by host’s enzymes) generated by the microbiome (201).

Gut microbiota produce and regulate multiple compounds that can reach distant organs *via* systemic circulation, and influence host physiology (202). More precisely, metabolic compounds produced or transformed by gut microbiota may modulate host immunity in distant sites. In particular, short chain fatty acids, especially butyrate, seem to exert broad anti-inflammatory activities by affecting immune cell migration, adhesion, cytokine expression, as well as cellular proliferation, activation, and apoptosis through the activation of signaling pathways (NF-κB) and inhibition of histone deacetylase (203). Moreover, epithelial permeability can be modulated by the microbiota: blood-brain barrier permeability is increased in germ-free mice and reintroduction of a healthy microbiota, of short chain fatty acids producing bacteria or direct short chain fatty acids administration can reverse this effect, up-regulating tight junction proteins expression (204). This mechanism could be involved at the host-environment interface, modulating pathogen susceptibility.

Fungi could also play a crucial role in long-reaching immune modulation. Digestive *Candida*-produced prostaglandin E2, an active lipid compound with hormone-like effects, can reach the lungs through the bloodstream, act on lung macrophages and promote allergic inflammation, thus suggesting a hypothetical long-reaching route that could be protective (205).

### Local to Local Protection

Some studies suggest that local microbiota could modulate distant local conditions, which may confer long-term protection to the host. On the one hand, few studies have highlighted a long-reaching protective role of specific local microbiota to specific local sites, and with poor mechanistic understanding. On the other hand, many papers have brought out various mechanisms that could involve distant modulations of immune conditions, but without a protective effect. Time has perhaps come to combine these approaches, to determine how the microbiota located in a given organ could have a protective effect on another organ (i.e., a local-to-local protection, but without a demonstrated systemic effect). Even though in some cases, later evidence might show that a local-to-local effect is actually a local-to-systemic effect, the conceptual distinction between a highly targeted mechanism versus a system-wide mechanism can guide research questions about the underlying mechanisms and the scope of protective effects. We explore two possible routes of long-reaching protection: the host-mediated gut–lung axis and direct interactions down the respiratory and gastrointestinal tracts.

#### Indirect, Immune-Mediated Protection along the Gut–Lung Axis

To the best of our knowledge, the only explicit long-reaching protective effects from one local site to another described in the literature are the immune-mediated relation between the gut microbiota and lung infections (203, 206). *Klebsiella pneumoniae* lung infection has been extensively studied as a model of pulmonary infection. Germ-free mice infected with *K. pneumoniae* are drastically susceptible to bacterial infection in an IL-10-dependent manner, with an increase of bacterial growth and dissemination. Interestingly, activating microbial pattern recognition receptors helps fight against *K. pneumoniae* infection in the lungs (207, 208). Another example is tuberculosis. *Helicobacter pylori* infection is suspected to modify the clinical outcomes of *M. tuberculosis* infection, with the presence of *H. pylori* associated with a lower rate of tuberculosis infection (209), but increased tuberculosis severity (210). In this case, a microbe at one location (*H. pylori* in the stomach) could modulate long-distance immunity at yet another location (in the lungs) in response to infection.

Antibiotic-treated mice exhibit impaired innate and adaptive mucosal immune responses to influenza infection, with increased damages and host mortality (179). A decrease in the number of gut commensals *via* treatment with a broad-spectrum antibiotic resulted in blunted T-cell and B-cell responses to an intranasal infection with the A/PR8 strain of influenza (211). Activation of Toll-like receptors restored the immune response in antibiotic-treated mice through inflammasome-mediated induction of interleukins such as IL-1β and IL-18. The authors suggest that either the microbial products can diffuse systemically, or activation of the inflammasome does not need to occur at the site of infection.

In an *E. coli* pneumonia model, antibiotic depletion of commensals in mice also causes a drastic bacterial burden both in lungs and blood, with a significant increase of mortality. Lipopolysaccharides supplementation during antibiotherapy reversed these effects, suggesting a distal action of commensal microbes through toll-like receptors (212). Deleterious effects of antibiotic depletion have been shown in a mouse model of *Streptococcus pneumoniae* infection, reversed by fecal microbiota transplantations that enhance macrophage functions in the primary alveolar (213).

Gut microbiota also play a role in expanding and maintaining viral-specific memory T-cell populations in the lungs. In a mouse model of MCMV-associated lung disease, the frequency of virus-specific CD8^+^ T cells in the MCMV-infected lungs of germ-free mice was restored by fecal bacteria from specific pathogen-free mice, likely through cross-activity between gut microbiota peptides and epitopes of MCMV-specific memory T cells (214).

Furthermore, mice challenged with pulmonary staphylococcal infection but lacking segmented filamentous bacteria in their gut microbiota showed more severe infection with higher bacterial load and mortality, associated with diminished lung concentration of Th17 immune effector cells. Reintroduction of segmented filamentous bacteria was sufficient to restore protective effect (215). Antibiotics also significantly decreased lung Th17 cell numbers during pulmonary acute fungal infection, restored by segmented filamentous bacteria colonization (216).

#### Direct, Ecological Protection through Digestive Flows and Air Circulations

Even though direct and host-mediated causes of microbial protection are oftentimes difficult to distinguish and intertwined at a local scale, the direct versus indirect distinction can help identify ecological versus physiological routes of influence at a global scale. Current evidence indicates that long-distance protection is mostly mediated by the host, but ecological routes are also available when we consider the way fluids and air flow through the host. Many organs are interconnected through airways and liquid channels. We hypothesize microbe-induced protection of downstream conditions in the respiratory tract and the gastrointestinal tract.

The respiratory tract ecosystem consists of the upper and lower respiratory tracts, with the latter further divided into trachea, bronchi, and bronchioles. The gastrointestinal tract ecosystem is divided into the stomach, small intestine, and large intestine, with secretions that come from associated organs such as the gall bladder, liver, and pancreas. It is possible that microbial communities in one patch of the lung and gut meta-communities can protect the host by influencing the entry and growth of pathogens in a downstream patch.

Bronchoalveolar lavage fluid samples of the lungs reveal a substantial microbiota community with multi-kingdom interactions due to air-borne fungi (217). The oral microbiota are the physiological source of lower respiratory tract microorganisms, predominately acquired through microaspiration and especially during sleep (218). It is also the source of disease-inducing pathogens in the lungs, especially pneumonia and cystic fibrosis. The sampled microorganisms of a healthy lung consist primarily of transitory populations determined by a steady balance of incoming migration and outgoing expulsion through physiological clearance or immune responses. In disease states, such as cystic fibrosis, regional growth conditions support the settled colonization and expansion of microbial communities (219, 220). The respiratory tract ecosystem is thus an excellent example of dominance in dispersal processes over processes of establishment and growth, similar to the metacommunity principles governing islands (220–224).

Microbial communities in the upper respiratory tract (e.g., oral cavity) are likely to confer resistance against the colonization and growth of pathogens in the lower respiratory tract. There is an association between oral dysbiosis and lower respiratory tract infections such as pneumonia. The absence of normal residents in the upper respiratory tract may thus contribute to the overwhelming migration of residential or opportunistic pathogens to the lower respiratory tract (225). Pathogen “blooms” in the lower respiratory tract generally occur under two conditions: when there is a positive influx of microorganisms over those eliminated or when altered local conditions favor pathogenic growth [see adapted island biogeography model in Ref. (223), and review in Ref. (224)]. We thus suspect that a loss of colonization resistance of upper respiratory tract residential microbiota may be the reason for lower respiratory tract microbial establishment and growth, resulting in disease.

Another possible downstream protective effect is in the gastrointestinal tract. The habitat of the gastrointestinal tract is similar to a river ecosystem (226). The intestine receives inputs from the source (nutrients and acids from the stomach) and associated organs (liver, gall bladder, and pancreas) and the flow slows down at the colon, which is dominated by greater bacterial loads and the accumulated nutrients and metabolites from upstream.

Secondary bile acids inhibit the germination and growth of *C. difficile* in the colon. Bile acids are released and mostly reabsorbed upstream in the small intestine, decreasing greatly in concentration further downstream (227, 228). However, small intestinal microbiota can inhibit re-absorption by deconjugating bile acids, thus promoting their excretion down and out of the large intestine (229). Downstream secondary bile acids in the colon can thus depend on upstream commensals. Indeed, antibiotic treatment that decreases colonization resistance against *C. difficile* also decreases bile acid amounts in fecal matter (64), which are both restored by fecal matter transfer (65). Upstream commensals can thus possibly protect the host from downstream pathogenic growth by releasing metabolite inhibitors.

Microbes can protect the host by regulating the migration flows between the lung “island” communities and by influencing “downstream” communities in the gut. The analogy between lung and gut ecosystems to island and river ecosystems, respectively, point to new perspectives. These are clearly local-to-local effects, although not necessarily organ-to-organ effects (they concern “subparts” of organs rather than organs *per se*). Yet they point to the possibility of organ-to-organ protective effects, and offer insights about how these effects could be investigated in the future. It is worth investigating whether migrations between other sites, for instance, the upper respiratory tract and the stomach (218), or direct dispersal between oral and vaginal microbiota *via* sexual behavior, also exhibit upstream-to-downstream colonization resistance against infections.

## Concluding Remarks and Perspectives

Previous studies have focused on the local colonization resistance conferred by microorganisms at various sites within the host. Here we examined protective effects beyond colonization resistance through both direct and indirect modes of protection. We highlighted how common types of ecological interactions give rise to the resistance and tolerance against harmful microorganisms as well as the containment of microorganisms, and how the innate and adaptive immune systems are activated by protective microbes, leading to resistance, tolerance, or containment toward pathobionts and pathogens. A better understanding of the range, mode, and effects of microbiota-mediated protection is crucial for therapeutic designs (Box 4). Although evidence for long-reaching microbe-conferred protection is scarce, we examined possible ways protective microbes can reach beyond their local sites, in part by investigating how pathogens spread their influence across the host.

Box 4 **Clinical perspectives about microbiota-mediated immunity.**

Clinical trials can be designed in two ways to test for the therapeutic effects of protective microorganisms: either look for yet unknown properties of well-known *in vivo* probiotics, or test the clinical effects of candidate microorganisms found in *in vitro* or animal models. Such trials are already underway. Based on *in vitro* studies of isolated oral commensals (230), a trial is designed to test the efficacy of oral probiotics to prevent ventilator-associated pneumonia (231). A pilot study has been conducted to test the tolerance of a nasal probiotic spray against upper respiratory tract infections (232). Microbe-conferred protection is important for host health and, as we have reviewed, can vary in range, mode, and effects. However, such protection may come at a cost for the host. Careful screening of not just protective effects but also of potentially harmful properties and long-term side effects are crucial, as well-known probiotics can exhibit genotoxicity (233) as well as inflammatory diseases and tumors (145).

Why is there so little evidence for long-reaching microbial protection? First, evidence for long-reaching protection is limited in part because microbiome research is still in its infancy. Therefore, despite growing interest in “protective microbes,” only very few detailed studies are available. Second, many protective mechanisms are localized actions. Classical dose-dependent toxicological effects (for instance, bacteriocin production) are unlikely to be involved in long-reaching mechanisms, as dilution volume is considerably higher in comparison with the production of local compounds. Third, technical limitations likely result in the underestimation of long-reaching protective microbes. Long-reaching effects rely probably more on low or even very low concentration mechanisms, with threshold effects (i.e., quorum-sensing molecules) that are extremely difficult to identify with classical shotgun metabolomics studies, thus requiring targeted studies. Another difficulty is the transitory release of long-reaching microbes and signals. For instance, bacteremia or fungaemia are brief events that are difficult to detect. Last, there are very few within-host ecological analyses in animal models. Yet the processes that regulate lung microbiota, for instance, show that we cannot understand respiratory disease without a dynamic ecological point of view.

We suggest a form of “co-immunity” between the host and microbiota. The host is protected against pathogens both by its own immune system and by the direct or indirect action of microorganisms, not only at a local scale but also between local communities and systemically.

It is generally assumed that immunity is the immunity of *one* organism, and that this immunity is bounded by the conventional frontiers of that organism (e.g., its skin). Yet it is now well-established that several immune phenomena transcend the boundaries of organisms (234). We suggest considering all these phenomena as instances of a more general process that we dub “co-immunity” (see also Box 2). We define co-immunity as a form of immune defense associating components of several organisms (it is, therefore, a “multi-organism” or “cross-organism” immunity). Co-immunity can include the protection of the young by maternal antibodies (235), microbiota-mediated immunity (as detailed in the present review), but also population-level phenomena such as social immunity in insects (236), and “herd immunity” (in particular through vaccination) (237, 238) in humans and cattle.

Rohwer and colleagues use the term “non-host derived immunity” to describe bacteriophage-mediated immunity (98). But this term also applies to immunity mediated by other components of the microbiota (not just bacteriophages) and, even more importantly, all these correspond to subcases of the wider category of what we call here “co-immunity.” This broad perspective helps us realize that the idea that immunity can transcend traditional boundaries of organisms is not as surprising as it might seem at first sight and is in fact certainly a widespread phenomenon in nature.

At the same time, the concept of co-immunity opens up many fascinating questions. One key question is to determine in which circumstances conflicts may arise between different components, and whether these conflicts are regulated. A microorganism that was protective at one moment can become detrimental to the host at another moment, for instance, in immunocompromised states (239). Similarly, maternal antibodies are generally protective for the infant, but they can inhibit the infant’s response to vaccination (240). Overall, a crucial advantage of the concept of co-immunity is that it reminds us that immunity should always be understood as a multi-actor and dynamic phenomenon.

## Author Contributions

LC and TB contributed equally to this paper. LC, TB, MT, LD, TS, and TP constituted all together the bibliography. LC, TB, and TP wrote the paper, which was substantially revised by MT, LD, and TS. LC was the primary author of the figures in this paper, with input and discussion from the other authors, especially TB and TP. TB was the main contributor to the clinical aspects. TP designed the project and managed the group.

## Conflict of Interest Statement

The author declares that the research was conducted in the absence of any commercial or financial relationships that could be construed as a potential conflict of interest.

## Footnotes

^1^Derek Skillings helped dubbed the term, noting its similarity to “community.”
